# Limited Coping Skills, Young Age, and High BMI Are Risk Factors for Injuries in Contemporary Dance: A 1-Year Prospective Study

**DOI:** 10.3389/fpsyg.2020.01452

**Published:** 2020-07-10

**Authors:** Diana van Winden, Rogier M. van Rijn, Geert J. P. Savelsbergh, Raôul R. D. Oudejans, Janine H. Stubbe

**Affiliations:** ^1^Codarts Rotterdam, University of the Arts, Rotterdam, Netherlands; ^2^Department of Human Movement Sciences, Vrije Universiteit Amsterdam, Amsterdam Movement Sciences, Amsterdam, Netherlands; ^3^Performing Artist and Athlete Research Lab (PEARL), Rotterdam, Netherlands; ^4^Institute of Brain and Behavior, Amsterdam, Netherlands; ^5^Faculty of Sports and Nutrition, Amsterdam University of Applied Sciences, Amsterdam, Netherlands; ^6^Rotterdam Arts and Sciences Lab (RASL), Rotterdam, Netherlands; ^7^Department of General Practice, Erasmus University Medical Center, Rotterdam, Netherlands

**Keywords:** performing arts, pre-professional, psychological, coping, perfectionism, self-regulation, injury

## Abstract

This study investigated potential risk factors (coping, perfectionism, and self-regulation) for substantial injuries in contemporary dance students using a prospective cohort design, as high-quality studies focusing on mental risk factors for dance injuries are lacking. Student characteristics (age, sex, BMI, educational program, and history of injury) and psychological constructs (coping, perfectionism, and self-regulation) were assessed using the Performing artist and Athlete Health Monitor (PAHM), a web-based system. Substantial injuries were measured with the Oslo Sports Trauma Research Center (OSTRC) Questionnaire on Health Problems and recorded on a monthly basis as part of the PAHM system. Univariate and multivariate logistic regression analyses were conducted to test the associations between potential risk factors (i.e., student characteristics and psychological constructs) and substantial injuries. Ninety-nine students were included in the analyses. During the academic year 2016/2017, 48 students (48.5%) reported at least one substantial injury. Of all factors included, coping skills (OR: 0.91; 95% CI: 0.84–0.98), age (OR: 0.67; 95% CI: 0.46–0.98), and BMI (OR: 1.38; 95% CI: 1.05–1.80) were identified as significant risk factors in the multivariate analysis. The model explained 24% of the variance in the substantial injury group. Further prospective research into mental risk factors for dance injuries with larger sample sizes is needed to develop preventive strategies. Yet, dance schools could consider including coping skills training as part of injury prevention programs and, perhaps, providing special attention to younger dancers and those with a higher BMI through transitional programs to assist them in managing the stress they experience throughout their (academic) career.

## Introduction

Dancers are high-performance athletes who are highly vulnerable to sustaining an injury (Ramkumar et al., 2016), which can have severe consequences, such as required medical treatment, experienced discomfort, decreased health-related quality of life, restricted artistic development due to absence from dance activities, and a significant delay in studying (Kenny et al., 2016; Yau et al., 2017; White et al., 2018). Previous literature showed prevalence and incidence rates of injuries among pre-professional ballet, modern and contemporary dancers ranging from 37 to 86% and from 0.77 to 4.71 injuries per 1,000 h of dance (Kenny et al., 2016; Lee et al., 2017; van Winden et al., 2019).

Insight into the etiology and mechanisms of injuries is of great importance to develop preventive measures and assess their effectiveness (van Mechelen et al., 1992; Finch, 2006). However, previous studies aiming at identifying risk factors for ballet and modern dance injuries have shown little consistence due to a lack of quality and level of evidence (Kenny et al., 2016). Moreover, dance medicine and science has traditionally focused on the physical aspects of injuries (Krasnow et al., 1999; Liederbach and Compagno, 2001; Mainwaring and Finney, 2017), while studies including dancers and athletes have shown that psychological constructs might also influence the occurrence of (dance) injuries and affect injury outcomes, including successful rehabilitation, duration of injury, and treatment-seeking behavior (Mainwaring and Finney, 2017; Reardon et al., 2019). Furthermore, mental skills can be considered adaptable (Kenny et al., 2016), which makes them appropriate for preventive measures.

The stress-and-injury model of Williams and Andersen (1998) suggests that psychosocial factors, namely, coping resources, personality, and history of stressors, influence the stress response and, in consequence, the probability of injury occurrence. More recently, the International Olympic Committee indicated in their consensus statement that limited coping resources and perfectionism are mental risk factors for injuries in sports (Reardon et al., 2019). Coping resources may help individuals to identify fewer situations and events as stressful, protecting them from stress and injuries (Williams and Andersen, 1998), whereas perfectionism can lead to increased stress levels, since performances are often viewed as an opportunity to fail rather than to succeed (Krasnow et al., 1999; Madigan et al., 2018).

Within ballet, there is some evidence that limited coping skills are associated with an increased injury risk (Noh et al., 2005). A quasi-experimental study showed that young ballet dancers who learned broad-based coping skills sustained less injuries and less severe injuries (i.e., shorter duration) compared with dancers who had not learned these skills (Noh et al., 2007). Furthermore, it is frequently believed that dancers are perfectionists (Nordin-Bates et al., 2011). Research has shown that injured ballet dancers scored significantly higher on perfectionism scales than non-injured dancers (Liederbach and Compagno, 2001). In addition, a study among modern and ballet dancers showed significant associations between perfectionism (i.e., high parental expectations, concern over mistakes) and injuries (Krasnow et al., 1999). However, these studies did not indicate the direction of the association or the causality due to methodological limitations, implying it is still unclear whether perfectionism can be seen as a risk factor for injuries in dance.

Furthermore, a recent study showed that high levels of self-regulatory skills (i.e., self-monitoring skills) can possibly help tennis players to prevent injury (van der Sluis et al., 2019). For instance, athletes/dancers could possibly prevent overuse symptoms from becoming time-loss injuries through the use of self-monitoring. However, to our knowledge, no study has investigated the relationship between self-regulation and injuries within a dance population.

Insight into risk factors for injuries enables us to develop preventive injury measures, which is of great importance considering the high frequency and disadvantageous consequences of injuries in dance. Furthermore, Kenny et al. (2016) stated that high-quality prospective cohort methods and multivariate regression modeling are needed within dance research to address causality, potential effect modification, and confounding factors. Therefore, the aim of this study is to establish whether psychological constructs (i.e., coping, perfectionism, and self-regulation) are potential risk factors for injuries in contemporary dance students. We hypothesized that limited coping skills, high perfectionism scores, and low self-regulation abilities would result in a higher risk of sustaining a substantial injury.

## Materials and Methods

### Participants

During a full academic year (September 2016–June 2017), 107 first year, second year, and third year contemporary dance students were prospectively followed. Students followed a 4-year educational program of either Bachelor dance or Bachelor dance teacher of Codarts Rotterdam, University of the Arts, the Netherlands. These programs contain modern technique classes (such as Cunningham and Graham), ballet classes, and contemporary classes (such as improvisation and partnering). Data was collected on a regular basis for management and educational purposes and embedded in the curriculum. Previously published longitudinal studies (van Winden et al., 2019; van Winden et al., in press), investigating the amount and characteristics of injuries and mental health problems, respectively, are (partly) based on the same sample. The present study utilizes a novel method, an analysis of potential psychological risk factors on the occurrence of dance injuries, which provides new interpretations of this data. All students were informed about the procedure and provided written consent in accordance with the Declaration of Helsinki. Ethical approval for the study was provided by the Medical Ethics Committee Erasmus MC of Rotterdam, the Netherlands (MEC-2019-0163). *A priori* power estimation has not been performed, since a pre-existing single cohort was available for this study.

### Measures

Student characteristics (age, sex, BMI, educational program, and history of injury), injuries and psychological constructs were assessed using the Performing artist and Athletes Health Monitor (PAHM) (Stubbe et al., 2018), a web-based system consisting of an extensive intake and a monthly follow-up, which includes the Oslo Sports Trauma Research Center (OSTRC) Questionnaire on Health Problems (Clarsen et al., 2014). This questionnaire has previously been used within performing arts (van Seters et al., 2017; Stubbe et al., 2018; van Winden et al., 2019).

#### Injury Registration

The OSTRC Questionnaire focuses on the consequences of health problems on participation, training volume, and performance along with the degree to which students perceive any symptoms. The four key items range from 0 (no problem/reduction/effect or no symptoms) to 25 (cannot participate at all or severe symptoms) (Clarsen et al., 2013). Questions 1 and 4 are scored on a four-point scale (0–8–17–25), while questions 2 and 3 are scored on a five-point scale (0–6–13–19–25). The OSTRC Questionnaire has a high internal consistency, with a Cronbach’s alpha of 0.96 and good face validity (Clarsen et al., 2013, 2014).

The severity of a health problem was calculated by the sum score of the four questions (scale 0–100) according to the method proposed by Clarsen et al. (2013). A health problem was registered when the severity score was higher than zero. The student was then asked whether the health problem was an injury, mental complaint, illness, or other complaint.

An injury was defined as “any physical complaint sustained by a dancer resulting in a severity score higher than zero (i.e., leading to consequences on participation, training volume, and/or performance), irrespective of the need for medical attention or time-loss from dance activities” (van Winden et al., 2019). Students were characterized as substantially injured if they reported problems leading to moderate or severe reductions in training volume (value ≥13 on question 2 of the OSTRC Questionnaire) or moderate, severe, or complete reductions in performance (value ≥13 on question 3 of the OSTRC Questionnaire) (Clarsen et al., 2014). Risk analyses were performed on substantial injuries, due to the more severe consequences and impact of these injuries on dance participation. Furthermore, preliminary results showed that less than 20% of the students were completely injury-free during the academic year, which would result in a very small reference group when all injuries were used as a dependent variable.

#### Psychological Constructs

The Athletic Coping Skills Inventory–28 (ACSI-28) (Smith et al., 1995) was used to measure general coping skills. The ACSI is a 28-item inventory of seven subscales: coping with adversity, coachability, concentration, confidence and achievement motivation, goal setting and mental preparation, peaking under pressure, and freedom from worry. The items are scored on a four-point Likert scale, ranging from 0 (almost never) to 3 (almost always). Total sum scores were calculated ranging from 0 to 84, with higher scores indicating greater coping skills. The ACSI has a test–retest reliability of 0.87 and a Cronbach alpha of 0.86 (Smith et al., 1995). The questionnaire was adjusted for the dance students, by replacing “coach or manager” with “teacher,” “competition” with “performance,” and “sports” with “perform.”

The dance-specific Perfectionism Inventory (Nordin-Bates et al., 2011) was used to measure perfectionism. This questionnaire is based on the Perfectionism Inventory (PI) of Hill et al. (2004), which was developed to include the multidimensional aspects of perfectionism within individuals in their daily lives. The dance-specific PI consists of seven scales: planfulness, striving for excellence, high standards for others, rumination, need for approval, concern over mistakes, and parental pressure. The first three subscales constitute the factor “conscientious perfectionism,” while the latter four result into the factor “self-evaluative perfectionism.” All items are rated on a Likert scale ranging from 1 (strongly disagree) to 5 (strongly agree). The dance-specific PI contains 51 items, with an internal reliability per subscale (Cronbach’s alpha values) ranging from 0.74 to 0.89 (Nordin-Bates et al., 2011).

Students completed the short version of the Self-Regulation Questionnaire (SSRQ) (Carey et al., 2004) to assess their capacity for self-regulation; that is, the ability to plan, guide, and monitor behaviors in the face of changing circumstances (Miller and Brown, 1991). The SSRQ is a 31-item questionnaire scored on a five-point Likert scale ranging from strongly disagree (1) to strongly agree (5). Sum scores were calculated, with higher scores indicating higher self-regulation capacity (Neal and Carey, 2005). The SSRQ has a Cronbach alpha of 0.92 and high correlation with the full-length SRQ (*r* = 0.96) (Carey et al., 2004; Neal and Carey, 2005; Hustad et al., 2009).

### Procedures

During the first month of the academic year (September 2016), baseline characteristics were recorded including age (years), body mass index [BMI; kg/m^2^, calculated from height (centimeters) and body weight (kilograms)], history of injury, coping, perfectionism, and self-regulation using the PAHM system. Injury history was defined as “any physical complaint resulting in a fulltime loss of dance activities (participation in class, rehearsal, performance practice, etc.) for at least 1 week beyond the day of onset in the past year” (van Seters et al., 2017), in accordance with the Fuller consensus statement (Fuller et al., 2006). Furthermore, during the academic year 2016/2017, injuries were recorded on a monthly basis. Only dance students who were injury-free at baseline and who completed > 30% of the monthly questionnaires were included in the analyses.

### Statistical Analyses

Statistical analyses were performed using SPSS Version 25 (IBM Corp., Armonk, United States), and statistical significance level was set at an alpha level of 0.05. Baseline characteristics were described using descriptive statistics, namely, mean and standard deviation (SD) or number and percentage (%). The incidence proportion of all injuries and substantial injuries was calculated by dividing the number of students that reported at least one injury or substantial injury during the academic year by the number of respondents in that same period (Knowles et al., 2006).

Univariate and multivariate regression models were used in order to look at potential injury risk factors. In addition to psychological constructs, student characteristics were taken into account, as a biopsychosocial approach toward injury risk management is desirable (Wiese-Bjornstal, 2010). Potential risk factors included age (years), sex (male), BMI (kg/m^2^), educational program (Bachelor dance teacher vs. Bachelor dance), and injury history in the previous year. Furthermore, the total coping score of the ACSI-28, the mean “conscientious perfectionism” and “self-evaluative perfectionism” factor scores of the dance-specific PI, and the total self-regulation score of the SSRQ were taken into account in the regression models. First, univariate logistic regression analyses between the potential risk factors and substantial injury (yes/no) were assessed to determine the relationship of each independent variable with the outcome variable. Second, all variables were included in a multivariate logistic regression model (method = enter). The results of the regression analyses for each potential risk factor were expressed in odds ratios (ORs) with corresponding 95% confidence interval (95% CI). Goodness-of-fit of the multivariate regression model was expressed in the Pearson chi-square statistic χ^2^, and the Nagelkerke R^2^ value was used to express the proportion of variance that was explained by the model (Field, 2009).

A total of 76 students (76.8%) provided complete data on all intake questionnaires (ACSI-28: 9 missing items; 0.33%, PI: 18 missing items; 0.36%, and SSRQ: 7 missing items; 0.26%), and 9 students had missing BMI scores (9.1%). Instead of relying on complete case analyses, multiple imputation via SPSS was used in order to increase the sample size (from *N* = 70 complete cases to *N* = 99). Only missing individual items within each questionnaire, as opposed to total scores, were imputed, as advised by Eekhout et al. (2014). Five imputed datasets were generated by SPSS, as recommended to be sufficient on theoretical grounds (Sterne et al., 2009). These five datasets were combined into one single set of pooled results for the regression analyses by SPSS. After data imputation, complete data was available for all 99 students on every measure.

## Results

### Response and Baseline Characteristics

A total of 137 students were enrolled in either the Bachelor dance or Bachelor dance teacher educational program, 134 students agreed to participate, and eventually the analyses were performed on 99 students (mean age: 19.2 ± 1.5 years). Twenty-seven students were excluded due to missing intake questionnaires, one student did not meet the inclusion criteria of a response rate higher than 30% on the monthly follow-up questionnaires due to dropping out of education for non-health related issues, and seven students were excluded because they were injured at baseline. Baseline characteristics of all included students are shown in Table 1. The response rate of the intake questionnaire was 78.6%. In total, 971 monthly questionnaires were sent to the students, and 822 were completed, resulting in a response rate of 84.7% for the monthly questionnaires.

**TABLE 1:** Baseline characteristics shown as mean (±SD) or number %.

	Overall
N	99
Education program (Bachelor dance)	55 (55.6%)
First year students	46 (46.5%)
Second year students	34 (34.3%)
Third year students	19 (19.2%)
Sex (male)	28 (28.3%)
Age (years)	19.2 ± 1.5
BMI (kg/m^2^)	20.9 ± 1.9
Dance exposure (total hours academic year 2016/2017 per student)	1,046.5

### Injuries and Risk Factors

A total of 80 students (80.8%) reported at least one injury during the academic year, while 48 students (48.5%) reported at least one substantial injury.

The univariate analyses showed no significant associations between the independent variables and substantial injuries during follow-up (see Table 2). However, a non-significant trend (*p* = 0.059) was visible for injury history (OR: 0.36; 95% CI: 0.12–1.04). Of all factors included, coping skills (OR: 0.91; 95% CI: 0.84–0.98), age (OR: 0.67; 95% CI: 0.46–0.98), and BMI (OR: 1.38; 95% CI: 1.05–1.80) were identified as significant risk factors in the multivariate analysis. The multivariate model resulted in a model with good fit to the data [χ^2^(*df* = 9) = 20.02, *p* < 0.05], indicating a good match between the specified model and the empirical data. In total, the model explained 24% of the variance in the substantial injury group.

**TABLE 2:** Univariate and multivariate models of potential risk factors for substantial injuries.

	Non-injured (*N* = 51)^*a*^	Injured (*N* = 48)^*a*^	Univariate analyses		Multivariate analyses	
			OR (95% CI)	*p*	OR (95% CI)	*p*
**Participant characteristics**						
Age (years)	19.45 (1.78)	18.94 (1.14)	0.79 (0.60–1.05)	0.10	0.67 (0.46–0.98)	0.04*
Sex (male)	17 (33.3%)	11 (22.9%)	1.68 (0.69–4.10)	0.25	2.23 (0.73–6.81)	0.16
Educational program (Bachelor dance)	28 (54.9%)	27 (56.3%)	0.95 (0.43–2.09)	0.89	0.80 (0.28–2.29)	0.67
BMI (kg/m^2^)	20.62 (1.77)	21.12 (2.03)	1.15 (0.93–1.43)	0.20	1.38 (1.05–1.80)	0.02*
Injury history (yes/no)	6 (11.8%)	13 (27.1%)	0.36 (0.12–1.04)	0.06	0.36 (0.11–1.24)	0.11
**Mental factors**						
Total coping score	52.78 (11.35)	49.14 (9.00)	0.97 (0.93–1.01)	0.08	0.91 (0.84–0.98)	0.01*
Mean score self-evaluative perfectionism	3.06 (0.44)	3.03 (0.33)	0.84 (0.31–2.34)	0.75	1.80 (0.42–7.72)	0.43
Mean score conscientious perfectionism	2.62 (0.48)	2.63 (0.41)	1.07 (0.44–2.59)	0.89	0.31 (0.07–1.30)	0.11
Total self-regulation score	114.18 (11.67)	111.1 (12.54)	0.98 (0.95–1.01)	0.21	1.01 (0.96–1.07)	0.66

OR, odds ratio; CI, confidence interval. ^*a*^Data are presented as mean (±SD) or number (%). *Significant at p < 0.05.

## Discussion

This is the first prospective cohort study investigating mental risk factors for substantial injuries among contemporary dance students. A 1-year substantial injury prevalence of 48.5% was found, meaning that “the students were not able to participate at all or had a moderate/severe reduction in training volume or performance because of an injury” according to Clarsen et al. (2014). Limited coping skills, lower age, and higher BMI were found to be associated with an increased injury risk. More specifically, per point higher on total coping score (range 0–84) students are 0.09 times (9%) less likely to sustain an injury, per year older students are 0.33 times (33%) less likely to sustain an injury, and per point higher on BMI students are 0.38 times (38%) more likely to sustain an injury. Furthermore, a non-significant trend was shown within the univariate analyses, suggesting students without an injury history are less likely to sustain an injury, which is in agreement with other studies in dance (Kenny et al., 2016) and in sports (Saragiotto et al., 2014; Alahmad et al., 2020; Green et al., 2020).

In total, the multivariate model explained 24% of the variance in the substantial injury group. However, the vast majority of sustained substantial injuries in this contemporary dance population was not explained by the factors included in this model. Therefore, future research within dance should investigate other risk factors as well. For instance, several studies have shown that joint range of motion (i.e., lower extremity), dance exposure (i.e., years of training, exposure hours), poor aerobic capacity (Kenny et al., 2016), and stress (Mainwaring and Finney, 2017) are associated with injury risk. These factors might illuminate part of the unexplained variance in the current multivariate regression model and should, therefore, be included in future studies.

### BMI

A recent systematic review within pre-professional ballet and modern dancers indicated that low BMI was significantly associated with an increased risk of injury (Kenny et al., 2016). However, this risk factor was based on only one, dated study (Benson et al., 1989), warranting further investigation. The present study showed that higher BMI (mean 20.9 ± 1.9) was associated with injury risk. Likewise, within sports, it has been shown that high BMI increases the risk for all sport injuries in adolescents (Richmond et al., 2013). They indicated an increased injury risk in obese adolescents compared to healthy adolescents of 34%, possibly due to “greater forces being absorbed through soft tissue and joints.” Within the current study, four students scored above the norm of healthy BMI (> 25, according to the World Health Organization, 2019), and these students were all substantially injured during the academic year. Therefore, results from the current study suggest that BMI could be monitored in student populations in relation to injury risk, and perhaps, specific attention might be desirable for students with a higher BMI. However, the variability of BMI between injured and non-injured students in the specific target sample is small. Therefore, the results should be interpreted with caution, and more research on specific BMI cut-off points within a dance population is needed to determine which students have an increased injury risk.

### Age

Previous literature has shown conflicting results regarding age as a risk factor. Two studies indicated that higher age was associated with the incidence of contemporary dance injuries (mean age non-injured: 16.4 ± 3.8 years vs. mean age injured: 18.9 ± 4.8 years) (Campoy et al., 2011) and ballet/modern dance injuries (mean age: 15.8 ± 1.0 years) (Luke et al., 2002). However, Askling et al. (2002) found no association between age (mean age: 21 years, range 17–25) and the occurrence of hamstring injuries among ballet and modern dance students, whereas Bronner et al. (2003) found, in line with the results of the current study (mean age: 19.2 ± 1.5 years), that younger professional modern dancers (mean age: 22 ± 4.4 years vs. 27 ± 1.7 years) experienced a greater number of injuries. They suggest that “young dancers may require a transitional program to assist them in managing the new stresses of professional dance,” i.e., stress they experience throughout their (academic) career (Bronner et al., 2003), which can also be helpful for the younger students in the studied population. Furthermore, within the sports injury literature, the adolescent growth spurt is commonly related to increased injury risk due to increased muscle-tendon tightness and decreased physical strength (Caine et al., 2008). For instance, the rate of growth in elite adolescent ballet dancers (mean age: 16 ± 1.6 years) is associated with an increased risk of lower extremity overuse injuries (Bowerman et al., 2014). This might also apply to the youngest students in the present study, since the studied population ranged from 16 to 25 years. Therefore, these students might be at a greater risk of sustaining an injury due to the adolescent growth spurt compared to the older, more mature, students. Another possible explanation is a bias due to left truncation, which is a common problem in cohort studies (Cain et al., 2011). Our sample consists of first, second, and third year students, whereby students in the second or third study year have higher values for age compared to the first year students. However, we can only include the students who survived so far. For example, students in the third year already survived the first and second year. Their classmates who dropped out in their first or second year were not included in our third year sample. However, personal communication with the dance departments revealed that only a small minority of the students drop out of the first or second year due to injuries (two students in 2016/2017). Therefore, we believe that the influence of left truncation on our results is rather small.

### Coping

In line with the results of the present study, previous research within dance and sports has shown a relationship between coping and injury risk. A meta-analysis from different sport populations showed a weak relationship between coping skills and injury rates [*r* = −0.07, 80% CI (−0.10, −0.03)] (Ivarsson et al., 2017), while a systematic review of pre-professional ballet and modern dancers showed that insufficient coping skills (i.e., freedom from worry, confidence, and negative dance stress) are significantly associated with an increased injury risk (Kenny et al., 2016). The use of problem-focused coping strategies such as planning and problem solving may protect ballet and modern dancers from sustaining injuries by buffering the effects of stress (Barrell and Terry, 2003). These skills can probably help in perceiving fewer situations as stressful/problematic, as a stressful situation can cause greater injury risk due to muscle tension and lack of focus or attention (Williams and Andersen, 1998). However, due to small sample sizes in the current study, it was not possible to look into the different subscales/strategies of coping. Future research should, therefore, take different coping strategies into account, as well as a possible moderating effect via stress on injury vulnerability.

### Perfectionism

Perfectionism is often mentioned as a personality trait of ballet and contemporary dancers, and associated with maladaptive characteristics (Nordin-Bates et al., 2011). However, in the current study, no significant association was found between perfectionism and the occurrence of injuries. In contrast, Krasnow et al. (1999) and Liederbach and Compagno (2001) showed associations between perfectionism and injury occurrence among modern and ballet dancers. However, different measurement methods and questionnaires were used in both studies compared to the present study. Furthermore, causality was not shown in both studies due to methodological limitations. Finally, the studied population of Krasnow et al. (1999) had a lower mean age compared to the studied population (15.5 ± 0.5 years vs. 19.2 ± 1.5 years), whereas adolescence is marked as a key period for the development of individual differences in perfectionism (Damian et al., 2013), making it difficult to compare these results. It is possible that the direct link between perfectionism and injury is not visible due to an indirect effect of perfectionism on the relationship between stress and injury. Ballet and contemporary dancers with perfectionistic tendencies show greater anxiety and lower self-confidence than other dancers (Nordin-Bates et al., 2011), which can influence the stress response and, as a result, the probability of injury occurrence (Williams and Andersen, 1998).

### Self-Regulation

Self-regulation has been linked to various well-being outcomes (Sanders and Mazzucchelli, 2013). However, within the present study, no relationship between self-regulation and injury occurrence has been found. To our knowledge, no study has investigated the relationship between self-regulation and injuries within a dance population. A recent study within sports, among young female tennis players, did show that self-regulatory skills can predict injuries (van der Sluis et al., 2019). The difference could be explained by the large difference in mean age of the studied population (12.4 ± 1.1 years vs. 19.2 ± 1.5 years) and by the use of different self-regulation questionnaires, making it difficult to compare these results.

Moreover, it is known that athletes are often conflicted between protecting their health and expanding the boundaries of their bodies’ capacities in order to achieve peak performance, especially in adolescence (van der Sluis et al., 2019). During adolescence, an increased predisposition is present toward short-term success despite possible long-term consequences, due to developmental cognitive processes, which, in turn, seems to be related to injuries (van der Sluis et al., 2017). Within ballet, contemporary, and modern dance, a culture of dancing through pain and injuries is well known (Luke et al., 2002; Gamboa et al., 2008; Cahalan et al., 2019), making it even more difficult to make the right decisions regarding your health. For instance, as stated by Rivera et al. (2012), “perceptions about how dancers are expected to work through pain play a significant role in whether they will choose to rehabilitate.” Yet, it is also acknowledged that athletes, such as elite runners, use self-regulation to keep track of their bodies, by monitoring their feelings and pain in order to achieve long-term goals (Brick et al., 2015). Furthermore, Clark and Zimmerman (2014) indicate how people use self-regulatory skills to manage health and to prevent or control diseases. This apparent contradiction between protecting your health and pushing limits in order to reach peak performance could possibly explain why the results in the present study did not lead to a significant association between self-regulation and sustaining an injury.

Furthermore, self-regulation could possibly have a modifying effect on stress and, therefore, on sustaining an injury, according to the stress-and-injury model (Williams and Andersen, 1998). For example, studies among students have shown that self-regulation capacity can predict levels of well-being, mental health functioning, and stress and, thus, the likelihood of injury occurrence (Park et al., 2012; Gagnon et al., 2016). Moreover, Baumann et al. (2007) showed that negative effects (e.g., reduced emotional well-being and more psychosomatic symptoms) of a stressful situation were only visible when individuals had low (affect) self-regulatory abilities (e.g., impaired ability to self-generate positive affect or reduce negative affect). More research into the relationship between self-regulation, stress, and injury within dance is necessary.

### Strengths, Limitations, and Recommendations

The major strength of the current study is the prospective cohort design of risk-factor analyses with monthly follow-up, resulting in a low recall bias and a greater understanding of injury causality. In addition, the response rate to the monthly questionnaire was high (85%), possibly due to the use of an online feedback system (van Winden et al., 2019). Moreover, to our knowledge, this is the first study to gain insight into mental risk factors for substantial injuries within contemporary dance students on a longitudinal basis.

However, there are some limitations to this study. First, the power of the analyses are rather small due to a limited sample size (*N* = 99), since only a limited cohort was available for longitudinal research. Therefore, the commonly used “rule of 10” could not be applied (9 potential predictors, 48 events), resulting in overfitting of the final model. This can bias regression coefficients in both positive and negative directions (Peduzzi et al., 1996), since low power increases the likelihood of producing a miss (Type 2 error) or false-positive (Type 1 error) (Schweizer and Furley, 2016). This urges us to be careful in drawing (firm) conclusions.

Second, we conducted logistic regression analyses, and one of the restrictions of these models is that the exposure (in our case, the number of hours each dance student participated in dance activities) must be about equal for every participant (Bahr and Holme, 2003). In sports, playing time can vary greatly between players in the same team. For example, match exposure is significantly higher in the best players of the team compared to the substitutes. In our sample, this is not the case because all students were enrolled in either the Bachelor dance or Bachelor dance teacher education program, and therefore, there were no major interpersonal differences in the scheduling of classes, rehearsals, or performances (van Winden et al., in press). Furthermore, drop-out may lead to large variations in exposure time. However, in our study, only one student dropped out, and this student was excluded from the analyses. Finally, injuries may affect the exposure time. For example, in Dutch male soccer players, match incidence was 32.8 per 1,000 player-hours, and the median injury time loss was 8 days (Stubbe et al., 2015). Therefore, injuries may significantly reduce exposure time. However, in dance students, injury incidence and time loss is much lower, 1.9 injuries per 1,000 h of dance activity and 5.7 days, respectively (van Winden et al., 2019). Therefore, reduction of exposure due to injuries is less of a problem in dance studies, compared to studies including athletes. Third, this study used self-reported outcomes for injury severity and psychological constructs, which produced subjective data and a lack of detailed diagnostic information. To gain more insight into possible differences in mental risk factors between injury types, the inclusion of a follow-up by medical health professionals during the data collection period is recommended in future studies, indicating detailed diagnostic information per injury. For instance, the difference between “acute” vs. “overuse” injuries is relevant, since there might be different mental risk factors for acute vs. overuse injuries (Pensgaard et al., 2018). Furthermore, not all used questionnaires are dance specific. The coping questionnaire is sport specific and has been made dance specific for this study; however, psychometric properties were not re-evaluated. Furthermore, self-regulation has been seen as a domain general concept (van der Sluis et al., 2019). Finally, when using questionnaires on psychological constructs, it is important to be aware of the possibility of a social desirability bias (Neal and Carey, 2005). In order to reduce the potential of social desirability, results from the questionnaires were not shared on an individual level with the students’ teachers in this study, since dancers might worry that their results will impact their participation in classes or performances, which has been mentioned before in regard to physical health in dance (Kenny et al., 2017) and mental health in sports (Bauman, 2016).

## Conclusion

This study aimed to identify psychological constructs as risk factors for substantial injuries in contemporary dance students according to the stress-and-injury model of Williams and Andersen (1998), specifically coping and personality traits (perfectionism and self-regulation). The results partly confirm our hypothesis, since students with limited coping skills as well as younger students and students with a higher BMI are at higher risk for substantial injuries, while no significant results were found for perfectionism and self-regulation. These results are based on a relatively small population; therefore, the results should be interpreted with some caution. More prospective research into mental risk factors for dance injuries, among this high-risk population, with larger sample sizes is needed to develop preventive strategies. Besides, it is important to include stress (as a potential mediator) in further research, since there are different indications that, for instance, the influence of coping and perfectionism on injuries could (partly) run through effects of stress on sustaining an injury. Furthermore, exposure to risk factors for (dance) injuries can be frequent and variable throughout dance participation (Meeuwisse et al., 2007). As such, potential risk factors, such as stress or coping, should be collected at regular intervals, just as the outcome data.

Still, for now, the current findings provide us with new information on the relevance of coping, age, and BMI in sustaining a dance injury, which might be used to enhance the prevention of injuries within contemporary dance students. For instance, as stated by Ivarsson et al. (2017) “including psychological-based training programs into other types of injury prevention programs (e.g., biomechanical, strength training) has the potential to reduce the risk of injuries.” More specifically, dance schools could consider including coping skills training as part of injury prevention programs in their curriculum and, perhaps, providing special attention to younger dancers and those with a higher BMI through transitional programs to assist them in managing the stress they experience throughout their (academic) career.

## Data Availability Statement

The datasets generated for this study are available on request to the corresponding author.

## Ethics Statement

The studies involving human participants were reviewed and approved by the Medical Ethics Committee Erasmus MC of Rotterdam, the Netherlands (MEC-2019-0163). The patients/participants provided their written informed consent to participate in this study.

## Author Contributions

DW performed the data collection, analyzed the data, and wrote the manuscript. RR assisted with the data collection, analyzing the data, and writing the manuscript. RO, GS, and JS initiated the study and contributed to the content of the article. All authors contributed to the article and approved the submitted version.

## Conflict of Interest

The authors declare that the research was conducted in the absence of any commercial or financial relationships that could be construed as a potential conflict of interest.
